# COVID-19: Current Challenges and Future Perspectives

**DOI:** 10.3390/tropicalmed7020016

**Published:** 2022-01-24

**Authors:** Peter A. Leggat, John Frean, Lucille Blumberg

**Affiliations:** 1 World Health Organization Collaborating Centre for Vector-borne and Neglected Tropical Diseases, College of Public Health, Medical and Veterinary Sciences, James Cook University, Townsville, QLD 4811, Australia; 2 School of Public Health, Faculty of Health Sciences, University of the Witwatersrand, Johannesburg 2000, South Africa; 3 Centre for Emerging Zoonotic and Parasitic Diseases, National Institute for Communicable Diseases, Johannesburg 2131, South Africa; johnf@nicd.ac.za (J.F.); lucilleb@nicd.ac.za (L.B.)

This Special Issue focuses on recent global research on the current coronavirus (COVID-19) pandemic. The disease is caused by a novel virus, severe acute respiratory syndrome coronavirus 2 (SARS-CoV-2) [1,2]. The International Committee on Taxonomy of Viruses (ICTV) named the virus SARS-CoV-2, as it is genetically related to the coronavirus responsible for the SARS outbreak of 2003 [2]. While related, the two viruses are quite different in their behaviour. At the time of submission for publication (7 January 2022), COVID-19, named by the World Health Organization (WHO) on 11 February 2020, had caused more than 296.5 million cases and over 5.5 million deaths with over 2.6 million new cases in the past 24 h [2]. The COVID-19 pandemic has greatly affected the capacity of health systems providing essential health care [1], but more than 9.195 billion vaccine doses have been administered as of 10 January 2021 [2]. There have been 22 papers published upon peer review acceptance in this Special Issue, including one editorial, twelve research papers [3,4,5,6,7,8,9,10,11,12,13,14], three review papers [15,16,17] and seven other papers [18,19,20,21,22,23,24], including one perspective, two case reports, one brief report, two viewpoints and one commentary. They each contribute to a much better understanding of COVID-19.

The contributions of these papers can be summarized as follows for the 12 research papers. The first of the research papers was a study of mortalities due to COVID-19 through a retrospective, observational study that included all inpatients from a major hospital in Nepal. Interestingly, 16% of patients also showed microbiological evidence of secondary infection [3]. The second of these studies was a cross-sectional survey exploring the knowledge and perceptions of healthcare workers (HCWs) regarding COVID-19 issues during the second wave of the pandemic, in four tertiary care hospitals in Greece. This study reveals some misconceptions and knowledge gaps in HCWs’ everyday practice, especially regarding hand hygiene and antimicrobial use in COVID-19 patients [4]. The third study looked at COVID-19 vaccination in those aged 75 years and older in Brazil. COVID-19 vaccines were highly effective in reducing the number of COVID-19-related deaths in over 75-year-olds [5]. The fourth paper studied persistent symptoms in post-COVID-19 patients attending a follow-up clinic at a tertiary care hospital in Nepal. Of these, 97 (82.2%) patients reported that they had at least one persistent/new symptom beyond two weeks from the diagnosis of COVID-19, including dyspnoea, fatigue, chest heaviness, and cough, emphasizing the need to extend the monitoring of symptoms after discharge [6]. The fifth of these papers was a study to examine the impact of the COVID-19 pandemic on tuberculosis (TB) and human immunodeficiency virus (HIV) management in Zimbabwe. Impacts were demonstrated on the management of both diseases [7]. The sixth of these papers was a similar study conducted in Malawi, where there were similar impacts of the COVID-19 pandemic on TB and HIV management [8]. The seventh of these papers was a similar study conducted in Kenya, where there were similar impacts of the COVID-19 pandemic on TB and HIV management [9]. The eighth was a case series of five clinically- and laboratory-confirmed COVID-19 patients from Bangladesh, who suffered a second episode of COVID-19 illness after 70 symptom-free days. The study suggested the need for COVID-19 vaccination and continuation of other preventive measures to further mitigate the pandemic [10]. The ninth of these papers was a case of *Plasmodium falciparum* malaria in a patient asymptomatically co-infected with COVID-19, which reinforced the need not to miss the diagnosis of malaria infection [11]. The tenth of these papers was a study looking for common respiratory viruses amongst HCWs with upper and lower respiratory tract infection symptoms in a major hospital in Manila, Philippines. Of these, 38 (13%) identified positive for another viral etiologic agent, compared with seven (2%) who were positive for COVID-19 [12]. The eleventh of these papers sought to estimate the seroprevalence of COVID-19 in a cohort of general practitioners working in Catania, Italy, after the first wave of COVID-19, which was low (3%) [13]. The last paper was a survey by the WHO Special Programme for Research and Training in Tropical Disease (TDR) and collaborators of health workers trained through the Structured Operational Research and Training IniTiative (SORT IT). This survey was designed to determine whether they were contributing to the COVID-19 pandemic response and, if so, to map where and how they were applying their SORT IT skills. It showed that investing in training ahead of such public health emergencies is vital [14].

There were three review papers in this Special Issue. The first of these was a review of the literature on the influenza pandemics of the 20th century, and of the coronavirus and influenza pandemics of the 21st century. There are remarkable similarities among them and it was concluded that the public health response to the COVID-19 pandemic constitutes the basis for delineating best practices to confront future pandemics [15]. The second was a review of the efficacy and safety of lopinavir–ritonavir (LPV/RTV) in COVID-19. This review did not reveal any significant advantage in the efficacy of LPV/RTV for the treatment of COVID-19 over standard care [16]. The third review was an evaluation of severity and mortality in COVID-19 patients with underlying kidney and liver diseases. This study found an increased risk of severity and mortality in COVID-19 patients with liver diseases or chronic kidney disease [17]. There were seven other papers in this Special Issue. The first was a perspective, examining how operational research can improve the delivery of established health interventions and ensure the deployment of new interventions as they become available, irrespective of diseases [18]. The second was a case report of two cases of heart transplantation with concomitant infections with SARS-CoV-2, with rapid progression to death due to Chagas disease and cytomegalovirus dissemination [19]. The third was a brief report, which evaluated faecal calprotectin (FC) concentrations in 25 in-patients with COVID-19 pneumonia without gastrointestinal symptoms. Of these, 21 patients showed increased FC and a strong positive correlation between FC and D-dimer [20]. The fourth was a viewpoint providing an overview of common and different findings for both malaria and COVID-19, with possible mutual influences of one on the other, especially in countries with limited resources [21]. The fifth was also a viewpoint providing an overview of the potential impact of COVID-19 on TB programs and disease burden, as well as possible strategies that could help to mitigate the impact [22]. The sixth was a case report involving a patient with severe COVID-19, who was treated with a non-weight-based dosage of tocilizumab to prevent the onset of a cytokine storm. It was concluded that tocilizumab played a substantial role in his ability to avert clinical decline, particularly the need for mechanical ventilation [23]. The last was a commentary that looked at the challenges faced due to the pandemic, and the steps taken to protect the safety of trial participants and the integrity of the trial in the STREAM Clinical Trial, which is the largest trial for MDR-TB [24].

The diversity of papers, the depth of the topics and the relative geographical reach of the authors in this Special Issue confirm the continued collective major interest in COVID-19. There are 223 contributors to the 22 papers published in this Special Issue, with affiliations in Europe, Africa, North America, South America and Asia-Pacific. This wide-ranging open access collection contributes to a much better understanding on the epidemiology, presentation, diagnosis, treatment, prevention and control of COVID-19. As the editors of this Special Issue, we trust that you find the content useful, as the authors are pleased to share their knowledge with an international audience.

We currently have another opportunity to update advances in this field through a second Special Issue, “COVID-19: Current Status and Future Prospects”. We encourage you to publish your work in, or propose a Special Issue for, *Tropical Medicine and Infectious Disease* (https://www.mdpi.com/journal/tropicalmed).

## Data Availability

Not applicable.
